# Association of molecular biomarkers expression with biochemical recurrence in prostate cancer through tissue microarray immunostaining

**DOI:** 10.3892/ol.2015.3556

**Published:** 2015-07-31

**Authors:** DING MA, ZHE ZHOU, BING YANG, QUN HE, QIAN ZHANG, XIANG-HUA ZHANG

**Affiliations:** 1 Department of Urology, Peking University Shougang Hospital, Beijing 100144, P.R. China; 2 Department of Urology, Peking University First Hospital, Beijing 100034, P.R. China

**Keywords:** biomarkers, biochemical recurrence, prostate cancer, tissue microarray

## Abstract

The aim of the present study was to investigate the prognostic role of metallothionein-2A (MT-2A), E-cadherin, interleukin-6 (IL-6), cyclin-E, proliferating cell nuclear antigen (PCNA) and B cell lymphoma (Bcl)-2 in the biochemical recurrence of prostate cancer (PCa) using tissue microarray immunostaining. Tissue specimens from 128 PCa patients who underwent radical prostatectomy were processed and transferred onto tissue microarrays. The clinicopathological parameters of PCa patients were also recorded. Following immunohistochemical examination of MT-2A, E-cadherin, IL-6, cyclin-E, PCNA and Bcl-2 expression in PCa specimens, association analysis of biomarkers expression with the biochemical recurrence of PCa was performed. The results revealed that the overall rate of biochemical recurrence was 30.5% (39/128) and the median biochemical recurrence-free time was 19 months (range, 6–35 months). The biochemical recurrence rates in low-, intermediate- and high-risk PCa classification were 14.8 (8/54), 38.7 (24/62) and 58.3% (7/12), respectively. Survival analysis demonstrated that a decreased biochemical recurrence-free survival rate was noted in PCa cases with positive MT-2A and cyclin E expression as well as those with negative E-cadherin expression (P=0.022, 0.028 and 0.011, respectively). Subsequent multivariate Cox analysis revealed that MT-2A [hazard ratio (HR)=2.01; 95% confidence interval (CI)=1.08–3.15; P=0.005], E-cadherin (HR=1.79; 95% CI=1.08–2.21; P=0.042) and cyclin E (HR=1.92; 95% CI=1.22–2.45; P=0.020) were independent predictors of the biochemical recurrence of PCa. In conclusion, the present study provided clinical evidence that evaluation of molecular biomarkers expression may improve clinical prognostic accuracy for the biochemical recurrence of PCa. Of note, the expression of MT-2A, cyclin E and E-cadherin may serve as independent predictors for biochemical recurrence of PCa.

## Introduction

Prostate cancer (PCa) is one of the most common types of cancer in the male population and has the second highest mortality rate worldwide, following lung cancer (1,2). To date, the survival of patients with PCa has been improved by the introduction of the serum prostate specific antigen (PSA) test and improvements in therapeutic methods (3,4). However, a subset of patients following surgical or radiation therapy may develop biochemical recurrence (PSA recurrence), which is an important determinant for poor prognosis of PCa patients (5,6). The conventional clinical prognostic factors, including serum PSA levels, Gleason score and pathological stage, have been associated with PCa progression, whereas their predictive role for biochemical recurrence of PCa remains controversial (7–10). Therefore, efforts should be made to discover novel biomarkers that may improve the clinical prediction of the biochemical recurrence of PCa.

Several molecular biomarkers have been reported to be involved in the tumorigenesis and clinical progression of PCa. As the major subtype of metallothionein (MT), MT-2A has several key properties, including the detoxification of heavy metal, protection of cells against damage induced by oxygen radicals and the regulation of cell proliferation (11). One previous study demonstrated the important role of MT-2A expression in PCa development through promoting cell proliferation (12). E-cadherin is known to be involved in the maintenance of epithelium integrity and cell adhesion; in addition, the downregulation of E-cadherin expression was suggested to contribute to tumor invasiveness and metastasis of PCa (13). Interleukin (IL)-6 is a cytokine that act as an autocrine and paracrine proliferation stimulator and may have a major role in the pathogenesis of PCa (14). In addition, cyclin E has a critical role in promoting cell cycle progression during cell proliferation; however, it was reported that increased cyclin E expression was closely associated with malignant cell proliferation in PCa (15). Furthermore, proliferating cell nuclear antigen (PCNA) is often used to reflect the activity of cell proliferation in PCa tissue (16) and anti-apoptotic protein B cell lymphoma (Bcl)-2 has been found to be overexpressed in PCa tissue, which is critical for PCa development and progression (17).

The aim of the present study was to evaluate the expression of different biomarkers regarding cell proliferation, cell adhesion and anti-apoptosis in PCa tissue using high throughput tissue microarray immunostaining. In addition, the present study aimed to analyze possible association of biomarkers expression with the biochemical recurrence of PCa.

## Patients and methods

### 

#### Tissue specimens and clinical data

Between January 2008 and December 2012, a total of 128 PCa patients who underwent radical prostatectomy at Peking University Shougang Hospital and Peking University First Hospital (Beijing, China) were included in the present study. These patients did not receive radiotherapy or chemotherapy prior to surgery and clinical follow up data was available. The median time of postoperative follow-up was 35 months (range, 12–60 months). All the PCa cases were stratified into the following three groups according to the criteria in 2010 National Comprehensive Cancer Network clinical practice guidelines (18) on PCa: Low-risk, ≤cT2a, PSA <10 ng/ml, Gleason score ≤6; intermediate-risk, cT2b, PSA10-20 ng/ml, Gleason score=7; and high-risk, ≥cT2c, PSA >20 ng/ml, Gleason score ≥8. Clinicopathological data including patient age, pathological stage (tumor-node-metastasis classification system) and surgical margin status, was also recorded. Following tissue collection, the specimens were fixed in 10% neutral-buffered formalin (Bestbio Biotechnology Co., Ltd., Shanghai, China) and embedded in paraffin (Yijie Biotechnology Co., Ltd., Shanghai, China). Pathological diagnosis was performed preoperatively and confirmed postoperatively. The present study received approval by the Clinical Research Ethics Committee of the Peking University Shougang Hospital. Written informed consent was obtained from all of the patients.

The end point of follow-up study was the time of biochemical recurrence and was defined as a serum PSA level ≥0.2 ng/ml on two successive measurements performed 24 h apart (19). The period of biochemical recurrence-free survival was defined as the time interval from the date of surgery to the first day of biochemical recurrence.

#### Tissue microarray construction

All the tissue microarrays were constructed as previously described (20). In brief, one tissue core (0.6 mm) was taken from an individual paraffin-embedded PCa specimen (donor block) and arrayed precisely into a new paraffin block (35×20 mm; Zhongshan Golden Bridge Biotechnology Co. Ltd., Beijing, China) using a custom-built precision instrument (ATA-27; Beecher Instrument, Inc. Silver spring, MD, USA). Following block construction, 4 µm sections were cut from the microarray blocks using a Leica microtome (Leica RM2135; Leica Instruments GmbH, Hubloch, Germany) to support the adhesion of array elements. Overall, the tissue microarray block contained 128 donor blocks from specimens of all 128 PCa patients. The presence of prostate tissue on the arrayed specimens was verified via hematoxylin-eosin (Solarbio Biotechnology Co., Ltd., Shanghai, China) stained sectioning for the identification of pathological features associated with PCa tissue.

#### Immunohistochemistry

Tissue microarrays containing consecutive 4 µm sections were used for immunohistochemical staining. Staining was performed using theavidin-biotin complex (ABC) immunoperoxidase kit (SP-9000; Zhongshan Golden Bridge Biotechnology Co. Ltd.). Prior to antigen retrieval, deparaffinized sections were treated with methanol containing 3% hydrogen peroxide (Zhongshan Golden Bridge Biotechnology Co. Ltd.) for 10 min. Sections were then boiled for 5 min in a 0.01 M sodium citrate buffer (pH 6.0; Zhongshan Golden Bridge Biotechnology Co. Ltd.) in a water bath for antigen retrieval. Following washing with 0.01 M phosphate-buffered saline (PBS; ZLI-9062; Zhongshan Golden Bridge Biotechnology Co. Ltd.), sections were blocked with blocking serum (Zhongshan Golden Bridge Biotechnology Co. Ltd.) for 10 min at 37°C. The sections were incubated at 4°C overnight with rabbit monoclonal antibodies directed against human MT-2A (1:200; Sc11377), E-cadherin (1:150; Sc7870), cyclin E (1:100; Sc481), PCNA (1:200; Sc7907) and Bcl-2 (1:150; Sc783) as well as mouse monoclonal antibodies directed against human IL-6 (1:50; Sc1265), which were all purchased from Santa Cruz Biotechnology, Inc. (Dallas, TX, USA). Additional negative controls lacking antibody were also used. The sections were then incubated with biotinylated goat anti-rabbit immunoglobulin G (SP-9000 Reagent B) for 20 min at 37°C and subsequently incubated with peroxidase-labeled streptavidin (SP-9000 Reagent C). Finally, 3,3′-diaminobenzidine (D12384; Sigma-Aldrich, St. Louis, MO, USA) was used as a chromogen and hematoxylin (ZLI-9609; Zhongshan Golden Bridge Biotechnology Co. Ltd.) was used as a counterstain. In order to determine the expression of these biomarkers in PCa cells, five microscopic fields were visualized in each core (Olympus BX40 microscope; Olympus Corporation, Tokyo, Japan). The percentage of cells with moderate staining was required in ≥10% to be considered as positive expression.

#### Statistical analysis

Statistical analyses were performed using the SPSS 13.0 software (SPSS, Inc., Chicago, IL, USA). Continuous variables were expressed as the mean ± standard deviations. Statistical analysis was performed independently using the Student's *t*-test, Fisher's exact test for any 2×2 tables and Pearson's χ^2^ test for non-2×2 tables. The Cox proportional hazards model was used to evaluate the association of conventional clinicopathological parameters and biomarkers with biochemical recurrence in univariate and multivariate analysis. Univariate biochemical recurrence-free survival was tested using the Kaplan-Meier analysis and with log-rank test for difference. P<0.05 was considered to indicate a statistical significant difference between values.

## Results

### 

#### Clinical data

Of 128 PCa cases, there were 54 low-risk, 62 intermediate-risk and 12 high-risk cases. The mean age of PCa patients was 67.2±10.3 years (range, 53–78 years), the mean preoperative serum PSA level was 13.8±8.9 ng/ml (range, 1.3–32.1 ng/ml), the mean Gleason score was 6.8±1.9 (range, 4–10) and the positive surgical margin rate was 15.6%.

In this study, the median biochemical recurrence-free time was 19 months (range, 6–35 months). The biochemical recurrence rate was 30.5% in all cases (39/128) and for the low-, intermediate- and high-risk classifications the biochemical recurrence rates were 14.8 (8/54), 38.7 (24/62) and 58.3% (7/12), respectively.

#### Expression of the different biomarkers in PCa specimens

**Figure 1.**
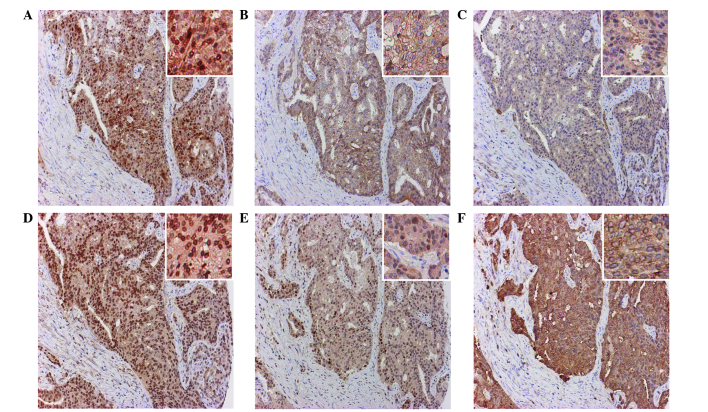
Representative immunostaining for MT-2A, E-cadherin, IL-6, cyclin E, PCNA and Bcl-2 in consecutive sections of a PCa specimen. Expression and location of different proteins in PCa cells: (A) MT-2A in cytoplasm and nuclei; (B) E-cadherin in the cytoplasm; (C) IL-6 in the cytoplasm; (D) cyclin E in the nuclei; (E) PCNA in the nuclei; and (F) Bcl-2 in the cytoplasm. Magnification, ×100; magnification of insets, ×200. MT-2A, metallothionein-2A; IL-6, interleukin 6; PCNA, proliferating cell nuclear antigen; Bcl-2, B cell lymphoma 2; PCa, prostate cancer.

The results of immunostaining for MT-2A, E-cadherin, IL-6, cyclin E, PCNA and Bcl-2 in PCa tissues are shown in Fig. 1. Expression rates of MT-2A, E-cadherin, IL-6, cyclin E, PCNA and Bcl-2 were 53.9 (69/128), 46.9 (60/128), 46.1 (59/128), 50.8 (65/128), 49.2 (63/128) and 53.1% (68/128) in PCa cases, respectively. Furthermore, the risk classification and pathological T (pT) stage of PCa were found to be positively associated with MT-2A (P=0.042 and 0.015), IL-6 (P=0.031 and 0.019) and cyclin-E expression (P=0.039 and 0.043), but inversely correlated with E-cadherin expression (P=0.032 and 0.012) (Table I). In addition, pT stage was positively correlated with PCNA and Bcl-2 expression (P=0.026 and 0.011, respectively).

#### Association of biochemical recurrence with clinicopathological parameters and biomarkers expression

According to the univariate and multivariate Cox regression analysis, PCa risk classification was demonstrated to be an independent risk factor for biochemical recurrence [hazard ratio (HR)=1.81; 95% confidence interval (CI)=1.56–2.19; P=0.047] (Table II).

**Figure 2.**
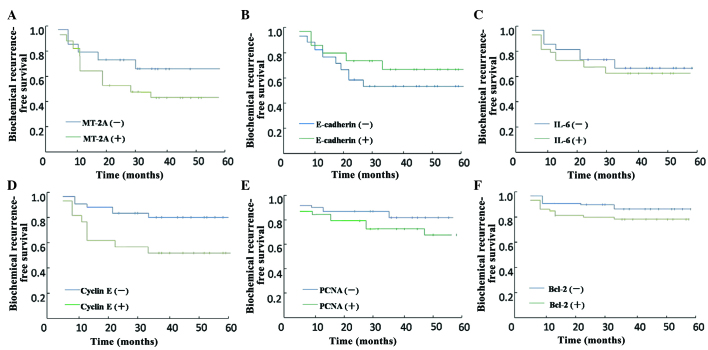
Kaplan-Meier method analysis of biochemical recurrence-free survival for prostate cancer patients. Correlations between: (A) MT-2A expression and biochemical recurrence-free survival (P=0.022); (B) E-cadherin expression and PSA biochemical recurrence-free survival (P=0.028); (C) IL-6 expression and biochemical recurrence-free survival (P=0.072); (D) cyclin E expression and biochemical recurrence-free survival (P=0.011); (E) PCNA expression and biochemical recurrence-free survival (P=0.091); and (F) Bcl-2 expression and biochemical recurrence-free survival (P=0.136). MT-2A, metallothionein-2A; IL-6, interleukin 6; PCNA, proliferating cell nuclear antigen; Bcl-2, B cell lymphoma 2.

Based on the results of univariate analysis, the positive expression of MT-2A (HR=2.15; 95% CI=1.14–3.08; P=0.009) and cyclin E (HR=1.45; 95% CI=1.02–1.92; P=0.037) as well as the negative expression of E-cadherin (HR=1.31; 95% CI=1.03–2.21; P=0.047) were indicated to be significant prognostic factors for the biochemical recurrence of PCa (Table II). Furthermore, univariate Kaplan-Meier/log-rank analysis revealed that decreased biochemical recurrence-free survival was noted in PCa cases with positive expression of MT-2A and cyclin E as well as negative expression of E-cadherin (P=0.022, 0.028 and 0.011 log rank test, respectively) (Fig. 2).

According to multivariate Cox regression analysis, MT-2A expression (HR=2.01; 95% CI=1.08–3.15; P=0.015), E-cadherin expression (HR=1.79; 95% CI=1.08–2.21; P=0.042) and cyclin E expression (HR=1.92; 95% CI=1.22–2.45; P=0.020) were demonstrated to be independent predictors for biochemical recurrence of PCa (Table II).

## Discussion

It has been established that the overall survival of PCa patients with biochemical recurrence may benefit from immediate adjuvant therapy following surgery; of note, conventional clinicopathological parameters were insufficient to predict biochemical recurrence accurately in certain cases (21,22). Through the tissue microarray immunostaining of six different biomarkers in PCa specimens, the results of the present study indicated that the expression of MT-2A, cyclin E and E-cadherin may provide a predictive role in the biochemical recurrence of PCa.

MT-2A and cyclin E have been previously reported to be important modulators involved in tumor cell proliferation (23,24). MT-1 and MT-2 have been studied in prostate carcinogenesis; the primary isoform of MT promoting malignant cell proliferation in PCa was identified as MT-2A (12,25,26). A previous report demonstrated a significant correlation of MT (including MT-1 and MT-2 isoforms) expression with Gleason score and biochemical recurrence in 70 PCa cases (27). Furthermore, cyclin E is known to promote cell cycle G1 to S progression during the process of malignant cell proliferation in PCa (15), whereas the correlation between cyclin E and clinicopathological parameters of PCa remains to be elucidated. The present study revealed a significant correlation of MT-2A and cyclin E expression with increased risk classification and pT stage in PCa. This may be due to stimulation of PCa cell proliferation via the MT-2A and cyclin E signaling pathways. In addition, the present results indicated that MT-2A and cyclin-E expression may be recognized as important factors for clinical progression of PCa.

Although clinicopathological parameters, such as risk classification and tumor stage, may be closely associated with the early biochemical recurrence of PCa (28), studies concerning the potential roles of MT-2A and cyclin E in predicting biochemical recurrence of PCa are limited. The present results revealed that decreased biochemical recurrence-free survival was closely associated with MT-2A and cyclin E expression in PCa. In addition, multivariate Cox analysis confirmed that MT-2A and cyclin E may serve as independent predictors for the biochemical recurrence of PCa.

The downregulation of E-cadherin expression in PCa tissue is critical for the initial steps of PCa invasion and metastasis (29). In concurrence with previous results (30), the present data revealed a close association of decreased E-cadherin expression with increased risk classification and pT stage in PCa. With respect to the association between E-cadherin expression and biochemical recurrence of PCa, previous studies revealed controversial results. Decreased E-cadherin expression was reported to be associated with increased biochemical recurrence rate in a cohort of 70 PCa cases (31), while E-cadherin expression failed to predict biochemical recurrence in another study of 82 PCa cases with Gleason scores of 5–7 (32). Through tissue microarray study in 128 PCa cases, the present results confirmed a close association between negative E-cadherin expression and decreased biochemical recurrence-free survival; this therefore indicated that E-cadherin may serve as an independent predictor for biochemical recurrence of PCa.

IL-6 has a critical role in PCa development through inducing malignant cell proliferation (16). Previously, the predictive effect of IL-6 expression in biochemical recurrence was reported in a cohort of 43 cases with advanced PCa (pT3/pT4) (33). The results of the present study demonstrated a significant correlation of IL-6 expression with the risk classification and pT stage of PCa; however, IL-6 expression was not found to be associated with biochemical recurrence according to Kaplan-Meier and multivariate Cox analyses. This result is not unexpected, as a larger number of organ-confined pT2 PCa cases were included in the present study; therefore, the predictive role of IL-6 in biochemical recurrence may be limited to advanced PCa. In addition, PCNA and Bcl-2 expression have been reported to be closely associated with tumor progression and poor overall survival in PCa (17,34). The present data confirmed a close association of PCNA and Bcl-2 expression with pT stage in PCa, whereas the predictive role of PCNA and Bcl-2 in the biochemical recurrence of PCa was not noted in the current study.

In conclusion, the present study revealed that the biochemical recurrence of PCa may be significantly associated with tumor cell proliferation status and loss of cell-cell adhesion, as reflected by MT-2A, cyclin-E expression and E-cadherin expression. Therefore, these results indicated that the evaluation of molecular factors may improve clinical predictions of the biochemical recurrence of PCa; notably, the expression of MT-2A, cyclin E and E-cadherin may serve as independent predictors for biochemical recurrence of PCa.

## Figures and Tables

**Table I.** Clinicopathological characteristics of prostate cancer cases according to biomarkers expression.

Characteristics	n	MT-2A (%)	P	E-cadherin (%)	P	IL-6 (%)	P	Cyclin E (%)	P	PCNA (%)	P	Bcl-2(%)	P
Age	128	61.8±6.5	0.469	61.9±5.8	0.452	65.9±9.0	0.512	67.1±8.3	0.396	64.2±7.1	0.602	65.8±6.3	0.476
Risk classification			0.042^a^		0.032^a^		0.031^a^		0.039^a^				0.136
Low-risk	54	25 (46.3)		20 (30.0)		22 (40.7)		22 (40.7)		30 (59.3)	0.299	28 (51.9)
Intermediate risk	62	36 (58.1)		33 (53.2)		30 (48.4)		35 (56.5)		28 (43.5)		35 (56.5)
High-risk	12	8 (66.7)		7 (58.3)		7 (58.3)		8 (66.7)		5 (33.3)		5 (41.7)
Pathological stage			0.015^a^		0.012^a^		0.019^a^		0.043^a^		0.026^a^		0.011^a^
pT2a	24	10 (41.7)		17 (70.8)		8 (33.3)		9 (37.5)		15 (62.5)		7 (29.2)	
pT2b	32	15 (46.9)		16 (50.0)		14 (43.8)		14 (43.8)		16 (50.0)		16 (50.0)	
pT2c	51	28 (54.9)		23 (45.1)		23 (45.1)		29 (56.9)		22 (43.1)		28 (54.9)	
pT3a	15	11 (73.3)		3 (20.0)		9 (60.0)		7 (46.7)		10 (66.7)		12 (80.0)	
pT3b	6	5 (83.3)		1 (16.7)		5 (83.3)		6 (100)		0		5 (83.3)	
Surgical margins			0.116		0.631		0.197		0.642		0.134		0.453
Negative	108	60 (55.6)		51 (47.2)		48 (44.4)		55 (50.9)		53 (49.1)		57 (52.8)
Positive	20	9 (45.0)		9 (45.0)		11 (55.0)		10 (50.0)		10 (50.0)		11 (55.0)

Values for age are presented as the mean ± standard deviation.

a P<0.05. MT-2A, metallothionein-2A; IL-6, interleukin 6; PCNA, proliferating cell nuclear antigen; Bcl-2, B cell lymphoma 2.

**Table II.** Univariate and multivariate Cox regression analysis for biomarkers expression and biochemical recurrence-free survival.

Analysis	Hazard ratio (95% confidence interval)	P-values
Univariate		
Risk classification	1.16 (0.91–1.69)	0.102
Pathological (pT) stage	0.99 (0.83–1.28)	0.189
Surgical margin status	0.81 (0.76–1.59)	0.203
MT-2A expression	2.15 (1.14–3.08)	0.009
E-cadherin expression	1.31 (1.03–2.21)	0.047
IL-6 expression	1.26 (0.98–1.85)	0.069
Cyclin E expression	1.45 (1.02–1.92)	0.037
PCNA expression	1.19 (0.89–1.83)	0.092
Bcl-2 expression	1.01 (1.02–3.15)	0.153
Multivariate		
Risk classification	1.81 (1.56–2.19)	0.047
Pathological (pT) stage	1.21 (0.96–1.93)	0.092
Surgical margin status	1.02 (0.65–2.12)	0.136
MT-2A expression	2.01 (1.08–3.15)	0.015
E-cadherin expression	1.79 (1.08–2.21)	0.042
IL-6 expression	1.65 (0.82–2.56)	0.062
Cyclin E expression	1.92 (1.22–2.45)	0.020
PCNA expression	1.34 (1.10–2.46)	0.075
Bcl-2 expression	1.21 (0.79–2.36)	0.103

MT-2A, metallothionein-2A; IL-6, interleukin 6; PCNA, proliferating cell nuclear antigen; Bcl-2, B cell lymphoma 2.
